# Supplementary material for: Glucose-Limited Fed-Batch Cultivation Strategy to Mimic Large-Scale Effects in Escherichia coli Linked to Accumulation of Non-Canonical Branched-Chain Amino Acids by Combination of Pyruvate Pulses and Dissolved Oxygen Limitation
Source: Microorganisms. 2021 May 21;9(6):1110. doi: 10.3390/microorganisms9061110 (PMC8223794; doi:10.3390/microorganisms9061110)
Supplement: Supplementary file 1 [file microorganisms-09-01110-s001.zip › Figure S2.pdf]

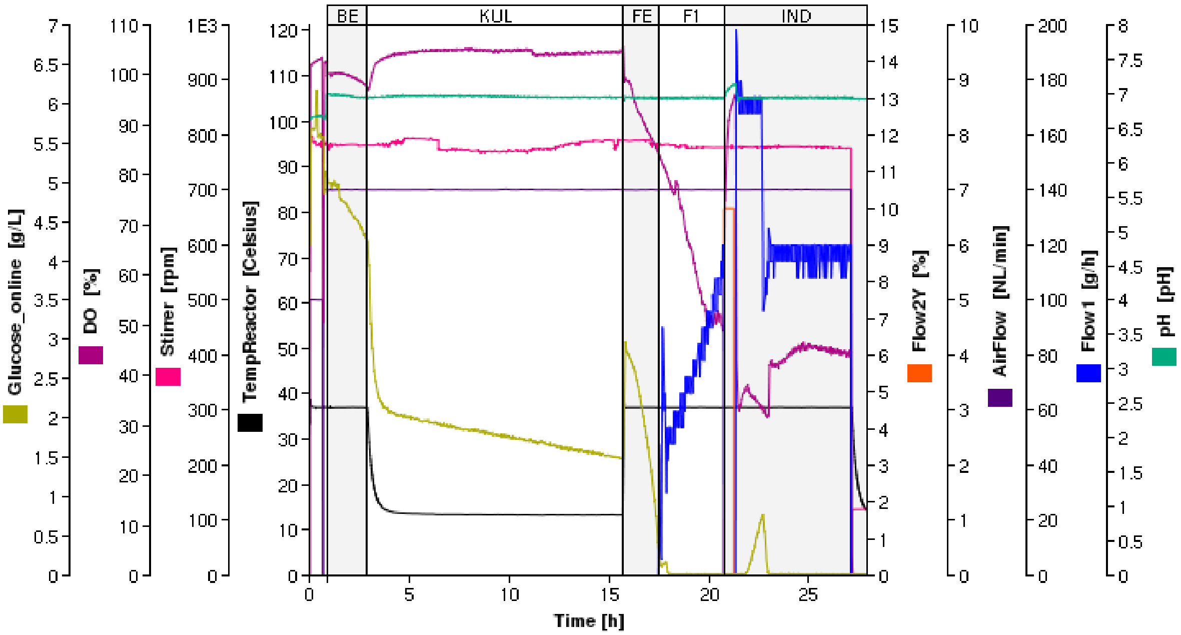

(a)

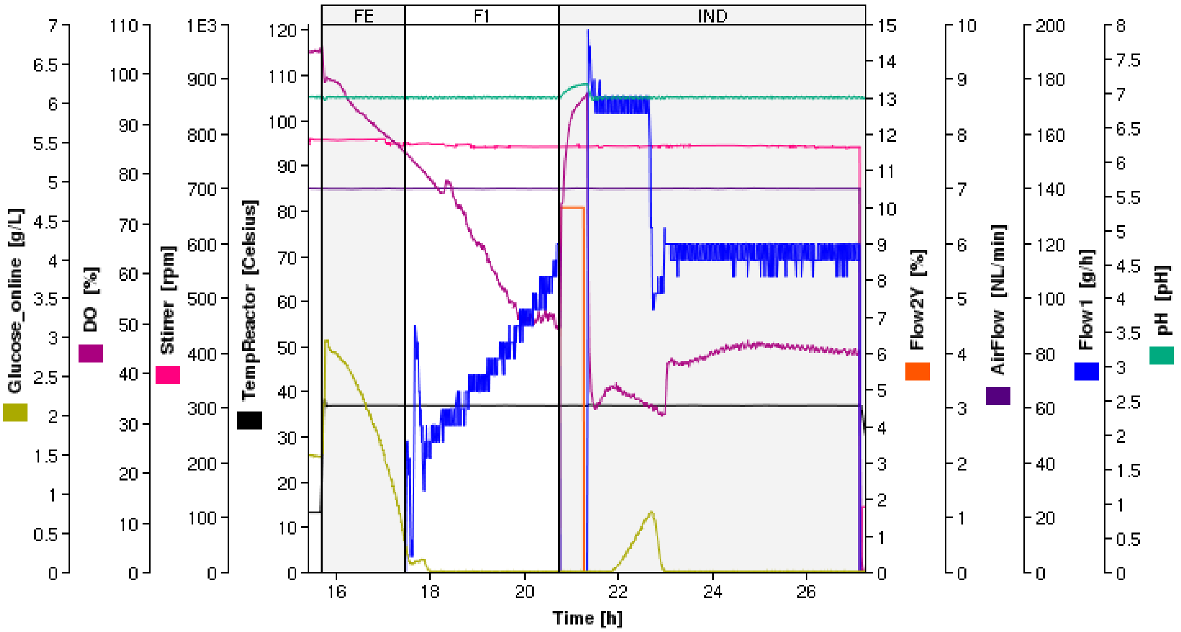

(b)

Figure S2. Overview of the reference cultivation of *E. coli* K-12 BW25113 pSW3_*lacI*+ in a 15L reactor, during the whole cultivation process (a) and during fed-batch period (b). Different cultivation phases are shown in the diagram as *BE* (first 2h of batch phase), *KUL* (13h cold period at 15 °C), *FE* (remaining 2h of batch phase), *F1* (3h exponential fed-batch phase) and *IND* (induction and linear fed-batch phase). IPTG induction was performed during 30 minutes (20.75 to 21.25 h cultivation time). Present in the diagram axes, *Flow1* corresponds to the flow rate (g/h) of the pump transporting the feed solution into the reactor while *Flow2Y* corresponds to one tenth of the flow rate (g/h) of the pump transporting the IPTG solution used for induction. During the linear feeding phase the constant flow rate was supposed to be equal to the last flow rate achieved in the exponential feeding phase; however, during the beginning of the linear fed-batch phase flow rate was unexpectedly higher. That explains the sudden accumulation of glucose (up to 0.8 g/L) between 22 and 23h cultivation time. Nevertheless, this was corrected by setting the flow rate to the proper value and glucose limitation was then again rapidly restored.
