# Supplementary material for: Glucose-Limited Fed-Batch Cultivation Strategy to Mimic Large-Scale Effects in Escherichia coli Linked to Accumulation of Non-Canonical Branched-Chain Amino Acids by Combination of Pyruvate Pulses and Dissolved Oxygen Limitation
Source: Microorganisms. 2021 May 21;9(6):1110. doi: 10.3390/microorganisms9061110 (PMC8223794; doi:10.3390/microorganisms9061110)
Supplement: Supplementary file 1 [file microorganisms-09-01110-s001.zip › Figure S3.pdf]

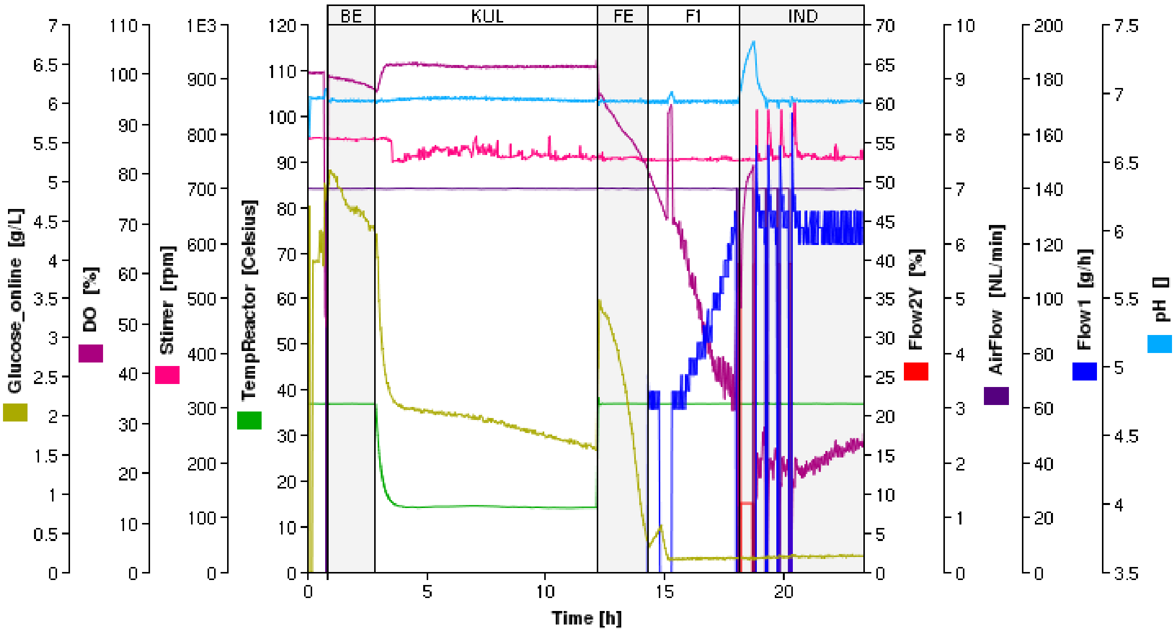
BE
KUL
FE
F1
IND
Glucose_online [g/L]
DO [%]
Stirrer [rpm]
TempReactor [Celsius]
Flow2Y [%]
AirFlow [NL/min]
Flow1 [g/h]
pH []
1E3
Time [h]

(a)

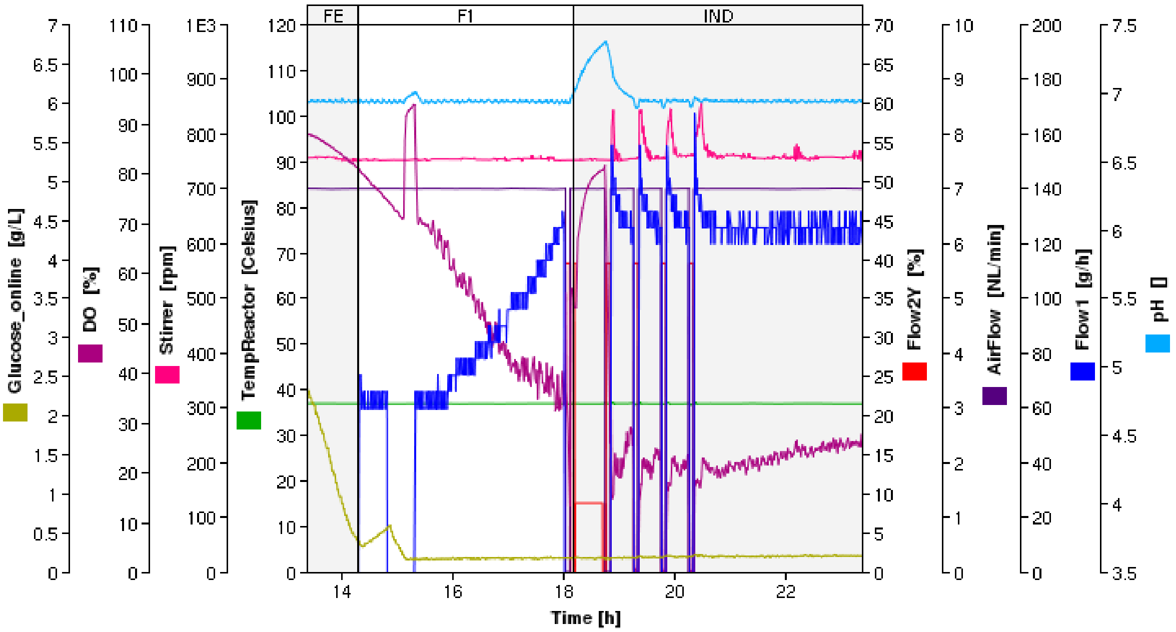
FE
F1
IND
Glucose_online [g/L]
DO [%]
Stirrer [rpm]
TempReactor [Celsius]
Flow2Y [%]
AirFlow [NL/min]
Flow1 [g/h]
pH []
1E3
Time [h]

(b)

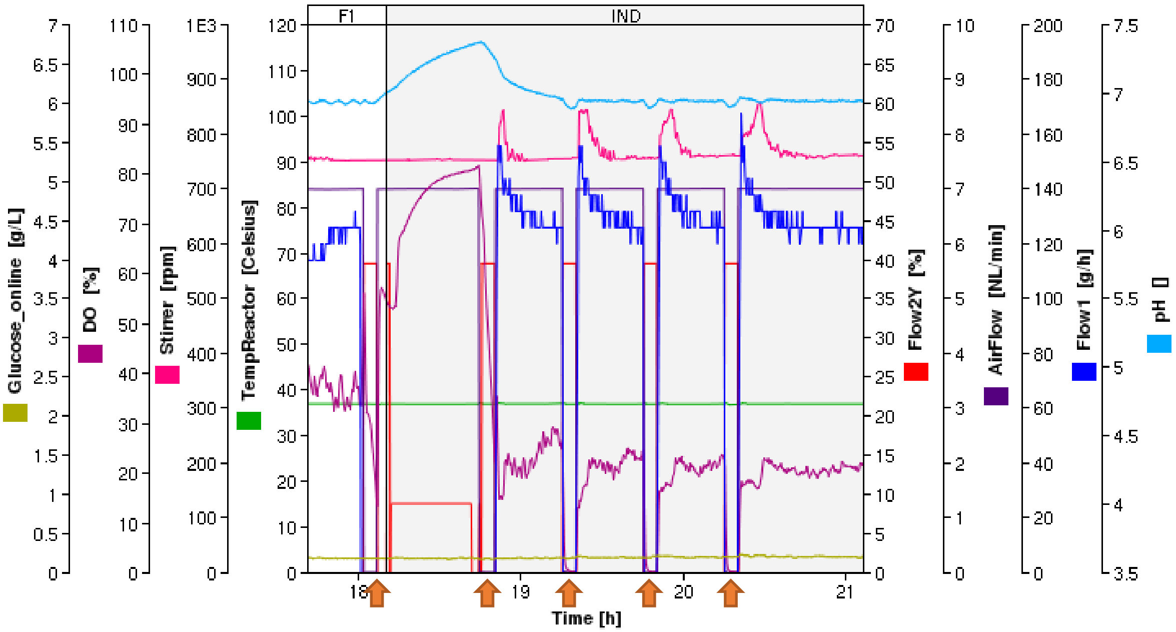

(c)

Figure S3. Overview of the cultivation of *E. coli* K-12 BW25113 pSW3_*lacI*+ exposed to pyruvate pulses and DO limitation in a 15L reactor, during the whole cultivation process (a), during fed-batch period (b) and during pyruvate pulsing (c). Different cultivation phases are shown in the diagram as *BE* (first 2h of batch phase), *KUL* (10h cold period at 15 °C), *FE* (remaining 2h of batch phase), *F1* (3h exponential fed-batch phase) and *IND* (induction, linear fed-batch phase and pyruvate pulsing). IPTG induction was performed during 30 minutes (18.25 to 18.75 h cultivation time). Pyruvate pulses are indicated by orange arrows (c). Present in the diagram axes, *Flow1* corresponds to the flow rate (g/h) of the pump transporting the feed solution into the reactor while *Flow2Y* corresponds to one tenth of the flow rate (g/h) of the pump transporting either the IPTG solution used for induction or the pyruvate solution employed for pulsing. Unexpectedly, exponential fed-batch started shortly before glucose was completely consumed in the batch phase. Hence, a small glucose accumulation was reported at around 15h. In order to correct this, exponential feeding was shortly shut down until glucose was completely depleted and then, activated again.
