# Supplementary figures and images for: Glucose-Limited Fed-Batch Cultivation Strategy to Mimic Large-Scale Effects in Escherichia coli Linked to Accumulation of Non-Canonical Branched-Chain Amino Acids by Combination of Pyruvate Pulses and Dissolved Oxygen Limitation
Source: Microorganisms. 2021 May 21;9(6):1110. doi: 10.3390/microorganisms9061110 (PMC8223794; doi:10.3390/microorganisms9061110)

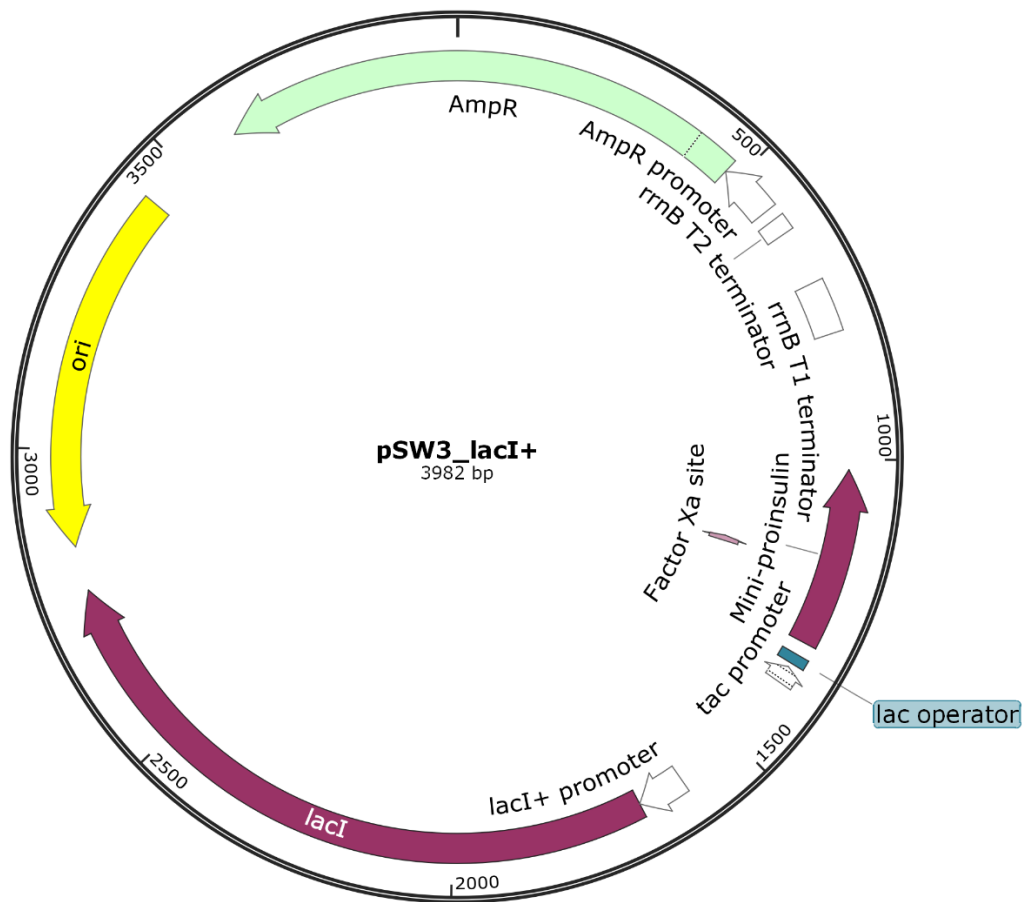

**Figure S1.** Genetic map of plasmid pSW3\_lacI<sup>+</sup>. Plasmid map was generated by Snapgene®.

Supplement: Supplementary file 1 [file microorganisms-09-01110-s001.zip › Figure S1.pdf]
